# Profiles of Cultural Adaptation and Parenting Approach for Childhood Obesity in Lifestyle Interventions for Families With Young Children: A Systematic Review

**DOI:** 10.1097/FCH.0000000000000397

**Published:** 2024-02-19

**Authors:** Ruben G. Fukkink, Yvonne S. Booij, Loes H. M. Leistra, Marloes D. A. van Verseveld

**Affiliations:** Research Institute of Child Development and Education, University of Amsterdam, Amsterdam, the Netherlands (Dr Fukkink and Ms Leistra); Centre of Expertise Urban Education, Amsterdam University of Applied Sciences, Amsterdam, the Netherlands (Dr Fukkink); Centre of Expertise Urban Vitality, Amsterdam University of Applied Sciences, Amsterdam, the Netherlands (Ms Booij); and Norwegian University of Science and Technology, Trondheim, Norway (Dr van Verseveld).

**Keywords:** cultural adaptation, early childhood, healthy lifestyle, obesity, parenting, systematic review

## Abstract

**Background and Objectives:**

Various interventions aim to reduce obesity and promote healthy lifestyles among different cultural groups.

**Methods:**

We have conducted a systematic literature review, following PRISMA guidelines (registered at https://doi.org/10.17605/OSF.IO/HB9AX), to explore profiles of cultural adaptation and parenting approach of lifestyle interventions for families with young children (1-4 years).

**Results:**

Our search (in CINAHL, ERIC, PsycINFO, PubMed, Scopus, and SSCI) yielded 41 studies reporting 31 interventions. Drawing on Intervention Mapping, we applied a newly developed framework with various indicators of cultural adaptation and a parenting approach to analyze interventions. Our review shows clear differences in the level of cultural adaptation. A categorical principal component analysis revealed 6 different empirical profiles of cultural adaptation.

**Conclusions:**

Based on our profiles, we discuss how cultural adaptation can be strengthened in the design of future early interventions aimed at promoting a healthy lifestyle.

CHILDHOOD OBESITY in the preschool period is a growing problem worldwide.^1^ The prevalence of overweight and obesity in preschool children increased from 4.2% in 1990 to 6.7% in 2010. According to the World Health Organization, there were 38 million overweight children younger than 5 years worldwide in 2019.^2^ Being overweight at an early age is a predictor for obesity in adulthood and is also associated with type 2 diabetes, hypertension, and cardiovascular disease later in life.^3^ Various interventions have been developed and evaluated for the prevention of childhood obesity. Reviews have shown that a combined approach with a center-based component and a home-based component contributes significantly to the effectiveness of these interventions.^4^–^6^ This implies that the culture of families and parenting styles in the domestic situation are important contexts for preventive healthy lifestyle interventions for children in their early years.

## CULTURALLY SENSITIVE LIFESTYLE INTERVENTIONS

Reviews in the field of healthy lifestyles and the prevention of overweight have emphasized the importance of culturally adapted interventions since parents' cultural background plays a role in their children's upbringing and feeding patterns,^6^,^7^ as well as obesity interventions for preschool children.^8^,^9^ Therefore, a “one-size-fits-all” approach to interventions is not sufficient.^10^–^12^ Cultural adaptation of interventions should increase their cultural fit^12^ to ensure that parents are more easily reached, identify more with the activities and are less likely to drop out, which in turn increases effectiveness.^13^–^16^ Cultural adaptation of interventions is defined as “systematic modification of an evidence-based treatment (EBT) or intervention protocol to consider language, culture and context in such a way that it is compatible with the client's cultural patterns, meanings and values.”^17^ Cultural adaptation may include adaptation to the focus community's language, culture and attitudes, and being attentive to specific needs. It can also include obtaining input from key figures within these communities to avoid mismatches with parents' prior knowledge and beliefs and stimulating acceptance, participation, and completion of the intervention. Surface structure adaptations^18^ involve changes in the materials or activities of the intervention that address observable and superficial aspects of a population's culture (eg, language, clothing, locations, and other observable aspects), whereas deep structure adaptations pertain to changes based on core value orientations, belief systems, and worldviews that influence the healthy lifestyle of a group.^19^ Cultural adaptation does not only involve modification of the program content but may also involve modifying the form of program delivery^18^ to reduce barriers to recruitment, improve program delivery by staff, and may strengthen the implementation in the local community environment.^12^ Finally, the adaptations may be related to cognitive-information-processing characteristics, such as language and age/developmental level, and to affective-motivational characteristics as related to gender, ethnic background, or socioeconomic status.^20^

An early review of 10 studies by Bender and Clark^8^ reported generally modest levels in the cultural adaptation of lifestyle interventions for families with preschool children, which fits in with other studies.^19^,^21^,^22^ Also, a recent review of 12 culturally adapted interventions for children 0 to 5 years of age by Marshall and colleagues^9^ concluded that cultural adaptation of childhood obesity interventions is modest. These 2 previous review studies, which have included only a relatively small number of studies, also emphasized the paucity of current studies and the need for further analysis.

## CULTURAL ADAPTATION FROM AN INTERVENTION MAPPING PERSPECTIVE

Several stage models have been proposed to guide the cultural adaptation of interventions,^21^,^23^,^24^ including Intervention Mapping (IM).^25^ IM was conceived originally as a method for developing interventions, but it also allows an analysis of the cultural adaptation of lifestyle interventions.^26^ The IM framework, which outlines explicit procedures and detailed conceptualization of program development, has been applied in various health contexts, including the cultural adaptation of community-based childhood obesity programs for multiethnic populations. The use of IM can provide health care planners thus with concrete guidelines for the cultural adaptation of programs.

IM distinguishes 6 steps in the planning of health promotion programs, starting with defining a logic model of the problem (1) and a model of change (2), followed by the design (3), production (4) and implementation of the program (5), and, finally, the evaluation (6). Distinguishing various concrete indicators of cultural adaptation for each IM step allows an in-depth analysis of cultural adaptation.

## PARENTING IN LIFESTYLE INTERVENTIONS

Parental sensitivity, structure, and control in everyday eating situations leave their mark on young children's feeding patterns and are a strong predictor of eating behavior later in life.^27^ General parenting styles and specific feeding styles and food-related parenting practices are associated with the cultural background of families.^7^ The theoretical model proposed by Sleddens and colleagues,^28^ which focuses on upbringing to achieve lifestyle-related behavioral change in families, puts, therefore, parenting at the heart of interventions. Some lifestyle interventions for families with children in their early years take into account general parenting styles of families (eg, authoritarian, authoritative, permissive, or uninvolved parenting)^29^ and/or specific feeding styles and food-related parenting practices (eg, parents' self-efficacy to provide healthy foods, providing rules/structure in meal setting and timing, explaining to child why healthy foods are important, promoting fun in healthy eating habits, and being a role model for child).^30^ However, we do not know yet whether parenting is an explicit part of different lifestyle interventions for families with young children.

## PRESENT STUDY

Research on cultural adaptation of healthy lifestyle interventions is still a relatively new field. Frameworks for cultural adaptation are still developing and guidelines are needed for (future) interventions for groups with diverse cultural backgrounds.^8^,^9^ Furthermore, previous review studies into cultural adaptation have not taken into account both the culture and parenting styles of families with young children,^27^ although the culture of families and their parenting are related. In a systematic review, we inventoried how healthy lifestyle interventions for families with young children (1-4 years) use a culturally sensitive approach and focus on strengthening parental skills and feeding practices. Building on previous reviews, which have generally shown relatively low levels of cultural adaptation in various fields,^8^,^9^,^14^,^15^,^19^,^20^,^22^,^23^ we focused in our study specifically on the identification of empirical profiles of cultural adaptation of healthy lifestyle interventions for families with children in the preschool period. In the context of our review study, Intervention Mapping^25^ offered a broad approach which makes it possible to categorize various elements of cultural adaptation in a systematic way from needs assessment, program development, and evaluation. With this analysis, we aim to explore in an empirical fashion current strategies of cultural adaptation, which may explain why the level of cultural adaptation is generally low for current interventions. We focused in our review on healthy lifestyle interventions for families with children in the preschool period from a prevention perspective; the first year of life was not included because this is a distinct developmental period for infants with the transitioning from milk feeds to family foods. Following the included studies from our review, we focused on ethnicity as the key marker for culture and cultural adaptation; in some cases, the focus on an ethnic group also implied that the families were part of a nondominant language community.

## METHODS

### Literature search

**Figure 1.**
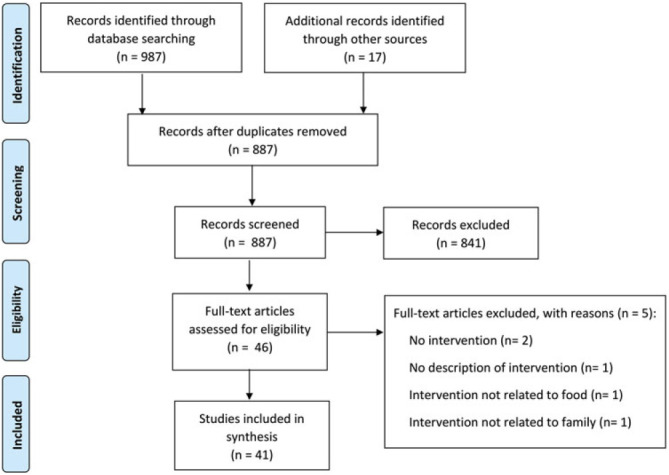
Flowchart of selection of studies.

The literature databases CINAHL (65 hits), ERIC (1), PsycINFO (1), PubMed (780), Scopus (24), and Social Sciences Citation Index (116) were searched for full-text, peer-reviewed, English language articles; the first search was conducted in May 2020 with an update in December 2021. This field has recently witnessed a rise of publications in the last decade and we searched for studies published since 2005. A broad search profile included key words in 7 domains: the parents, the child, parenting, interventions, lifestyle and weight status, nutrition and diet, and cultural sensitivity (see the Figure 1; Supplemental Digital Content Appendix 1, available at: http://links.lww.com/FCH/A68).

The abstracts from the search yielded 987 hits; we found another 17 studies by searching through the references from the publications.^31^ These abstracts were screened using 4 inclusion criteria. First, the study had to describe the development, implementation, and/or evaluation of an intervention. Second, it had to focus on the parents and/or extended family of children aged 1 to 4 years, with at least half of its age range coinciding with this group (so a study of children aged 2-6 years, eg, did fall within this criterion). Third, the intervention had to be aimed at preventing or reducing overweight or obesity, with a focus on nutrition (optionally supplemented with attention for sleep and/or exercise). The final criterion was that only primary studies were included. The most frequent reason for exclusion was that the age range of the children did not match with our population criterion.^32^ Other studies were excluded because the intervention did not match our criteria^33^ or the intervention was directed only at health outcomes for parents and not children.^34^ In case of any doubt after reading the abstract, the full article was read and discussed by the research team.^35^ This procedure resulted in a hybrid sample of 41 studies (ie, experimental evaluations of interventions, studies with a focus on implementation, qualitative studies, design papers) reporting on 31 interventions with a shared focus on healthy lifestyle interventions.

We followed the PRISMA 2020 guidelines and the review protocol was registered with Open Science Framework (https://doi.org/10.17605/OSF.IO/HB9AX). Only coding the quality of the included studies was not part of our study, because our sample of studies was highly diverse and the quality of studies was not related to our research questions.

#### Coding

The selected studies were coded using a newly developed scheme (see Supplemental Digital Content Appendix 2, available at: http://links.lww.com/FCH/A69), which was tested in 3 calibration sessions with independent assessment by all the authors. The rationale of the scheme that we developed was the need for a broad overview of concrete ways to culturally adapt an intervention, taking into account both the culture and the parenting style of diverse families with young children. We wanted to integrate the different features in a stage model and theoretically structured our coding scheme with the use of Intervention Mapping,^25^ including various concrete features of cultural adaptation from the literature. These variables were coded as present or absent and this dichotomous scoring method allows the calculation of an overall score to analyze and compare both the cultural adaptation and the parenting approach of healthy lifestyle programs.

The coding scheme was divided into 4 parts: description of the study (eg, type of report; study design; age of children; and weight status of the children); general characteristics of the program; cultural adaptation of the program; and parenting approach of the program.

The programs were classified according to the following features: primary focus—parenting skills in general; parenting practices related to nutrition, healthy eating habits, exercise, and other physical activity and sleep^36^; mode of delivery^37^—individual or group, face-to-face, or other (eg, email, telephone); focus group—child, mother, father, both parents, extended family, community, and early childhood professionals^36^; delivery agent (eg, child health clinic staff, nurses, community health workers, pediatrician, and staff from early childhood education and care)^38^; and the setting (eg, community, clinic). Programs were further classified according overall duration, number of sessions, and their length.

The cultural sensitivity of the intervention was coded using a coding scheme with 53 categories, classified on the basis of the 6 IM steps. The indicators for step 1 from the IM model (ie, “logic model of the intervention”) included the adaptation of intervention goals (often referred to as “tailoring” or “framing” in the included studies). The indicators related to IM step 2 (“logic model of change”) pertained to the selection of culturally appropriate determinants for a healthy lifestyle and the inclusion of culture-specific behavioral outcomes. For example, we coded whether an intervention explicitly considered “folk health beliefs” or cultural-specific “funds of knowledge,” defined as intellectual and cultural resources from families to strengthen the connection between families' homes and the intervention.^39^ The indicators related to step 3 (“program design”) related to adding new, culturally appropriate modules to the original intervention, any cooperation with the community during the program design, and the choice of culturally appropriate methods concerning the logic model of change,^37^ such as the translation of materials, the use of simplified language, and the adoption of alternative formats (eg, pictures or pictograms). Under step 4 (“program production”), we coded whether the focus community was explicitly taken into account when creating or adjusting program materials. For step 5 (“program implementation”), we coded the following features: identification of the program's first users within a particular group; referral of families via community and peer-to-peer networks; staff screening and training in respect of so-called “inclusion competencies”^8^; and the matching of staff with families based on language or ethnicity. Barriers identified in the literature^26^,^38^ were also included here (eg, low literacy skills of parents), because they may complicate the implementation of the program. Finally, for step 6 (“evaluation”), we coded whether cultural diversity of the target group was taken into account in the final evaluation phase (eg, by employing a diversity audit, coevaluation with stakeholders, or analyzing cultural background as moderator). We calculated a Cultural Adaptation score (CA), expressed as a percentage of a total of the 53 dichotomously scored CA variables (Cronbach α = .90). We coded each variable as “present” or “absent.” The percentage score was computed from the number of present variables, divided by the total of possible indicators (N = 53).

With regard to the parenting approach of the interventions, we coded whether the publication referred explicitly to the classic parenting styles (authoritarian, authoritative, permissive/indulgent, and uninvolved)^29^ or specific feeding styles and food-related parenting practices,^28^,^29^ distinguishing between nurturance, structure, behavioral control, overprotection, and coercive control. Each intervention was classified according to the continuum proposed by Dunst et al,^40^ which ranks interventions from “professional-centered” to “family-allied,” “family-focused,” and “family-centered.” In professional-centered interventions, for example, professionals lead as experts who determine what the family needs. Conversely, family-centered interventions are characterized by a flexible approach adapted to the specific needs of the family concerned. The parenting variables were summarized in a Parenting Approach score (PA), expressed as a percentage of a total of 28 dichotomous variables (α = .82). We coded each parenting variable as “present” or “absent.” The percentage score was calculated as the total of present variables, divided by the total of possible variables (n = 28).

We obtained all our information about the characteristics of Cultural Adaptation and Parenting Approach from the published articles. In a random sample of the interventions (20%), studies were coded by all possible pairs of 4 coders (ie, first author with second author; first with third; first with fourth; second with third; second with fourth; and third with fourth) to assess coder agreement for CA and PA scores. For all pairs, reliability was acceptable for CA and excellent for PA (average intraclass correlation coefficient [ICC], 2-way mixed, absolute agreement = .688 and .909, respectively). Discrepancies were resolved in sessions between the pairs of independent coders.

### Analysis

Interventions described in multiple studies were aggregated into a single intervention description; interventions were the unit of analysis in our study. Using categorical principal components analysis (CATPCA),^41^,^42^ we subsequently explored whether there were distinct profiles in the cultural adaptation of interventions. CATPCA is a flexible statistical technique for dimension reduction with minimal loss of information. In our study, we applied this technique to identify dimensions of cultural adaptation, based on a statistical analysis of the coded studies. This technique is suitable for handling discrete data and takes into account the dichotomous (ie, categorical) nature of all coded variables from our multifaceted coding scheme. Based on the 6 IM steps, we explored a 6-dimensional model with varimax rotation and Kaiser normalization. Four IM items and the other indicators were not included in this analysis because there was little or no variance due to very low scores.

## RESULTS

Table 1 presents an overview of the included studies and Table 2 gives information about the interventions. The 41 studies included were either evaluation (N = 28) or design studies (N = 13), mostly conducted in the United States, United Kingdom, and other countries. A slight majority (54%) involved parents from different ethnic backgrounds, while in a fifth, studies focused on a specific ethnic group (20%). Parenting styles were not explicitly addressed in most studies.

**TABLE 1.** Characteristics of the Included Studies^a^

Study	Country	Program	Setting(s)	Professional(s)	Type of Study
Alkon et al^43^ (2014)	United States	NAP SACC Intervention & Raising Healthy Kids	ECEC	Health nurse	Evaluation
Barkin et al^44^ (2018)	United States	GROW	Community	...	Evaluation
Beckerman et al^45^ (2019)	United States	CHL program	Online Clinic	Health nurse	Design
Bender et al^46^ (2013)	United States	Vida Saludable (Healthy Living)	Clinic	Health nurse	Evaluation
de Bourdeaudhuij et al^47^ (2015)	8 EU countries	IDEFICS	ECEC Home Community	...	Evaluation
Bridge et al^48^ (2019)	United Kingdom	HENRY	Clinic	Health nurse	Evaluation
Burton et al^49^ (2019)	United Kingdom	HENRY	Clinic	...	Evaluation
Buscemi et al^50^ (2016)	United States	Hip-Hop to Health	ECEC	ECEC staff	Evaluation
Cloutier et al^51^ (2015)	United States	Steps up to growing healthy	Telephone Clinic	Health nurse	Evaluation
Davison et al^52^ (2013)	United States	CHL program	ECEC	...	Evaluation
Dickerson et al^53^ (2016)	United Kingdom	Born in Brandfords Better start (BBB)	Clinic Community	...	Design
Ek et al^54^ (2020)	United Kingdom + Sweden	More and Less ML study	...	...	Evaluation
Greenmills et al^55^ (2013)	United States	Communities For Healthy Living: Communication Campaign	ECEC	Researcher	Design
Hacioglu and Simsek^56^ (2019)	Turkey	...	...	...	Evaluation
Haines et al^57^ (2013)	United States	Healthy Habits, Happy Homes	Telephone Home	Health nurse	Evaluation
Heerman et al^58^ (2018)	United States	COACH	Community	...	Design
Ingalls et al^59^ (2019)	United States	FSN	Home	...	Evaluation
Keita et al^60^ (2014)	United States	Healthy Habits, Healthy Families (HHHF)	Telephone Home	...	Evaluation
Knierim et al^61^ (2018)	United States	The COOT program	Home	...	Evaluation
Lebron et al^62^ (2020)	United Kingdom	CCC	ECEC	ECEC staff	Evaluation
McGarvey et al^63^ (2006)	United States	...	Clinic	...	Design
**Study**	**Country**	**Intervention**	**Setting(s)**	**Professional(s)**	**Type of Study**
McKee et al^64^ (2010)	United States	FLAIR (pilot)	Clinic	Pediatrician Lifestyle coach	Evaluation
Messiah et al^65^ (2017)	United States	Healthy Caregivers—Healthy Children (HC2)	ECEC	ECEC staff	Design
Montana et al^66^ (2015)	United States	Family Check-Up	Home	...	Evaluation
Natale et al^67^ (2013)	United States	Healthy Caregivers—Healthy Children (HC2)	ECEC	Dietician	Design
Po'e et al^68^ (2013)	United States	GROW	Community	...	Design
Salvy et al^69^ (2018)	United States	HABITS	Home	Family coach	Design
Sherwood et al^70^ (2013)	United States	NET-works	Home Clinic	Health nurse Pediatrician Family coach	Design
de Silva-Sanigorski et al^71^ (2010)	Australia	Kids Go For Your Life	ECEC Community	Family coach	Design
Smith et al^72^ (2015)	United States	Family Check-Up	Home	...	Evaluation
Sosa et al^73^ (2016)	United States	Miranos!	ECEC Home	ECEC staff	Evaluation
Sun et al^74^ (2017)	United States	5-4-3-2-1-0 Program	Home	Researcher	Evaluation
Taverno Ross et al^75^ (2018)	United States	ANDALE Pittsburgh	Home	...	Design
Taverno Ross et al^76^ (2017)	United States	ANDALE Pittsburgh	Home Community	...	Evaluation
Thomson et al^77^ (2014)	United States	PaT control arm & PaTE intervention arm	Home	...	Evaluation
Wickel et al^78^ (2019)	United Kingdom	The ELI clinic	Clinic	Health nurse Psychologist Dietician	Evaluation
Williams et al^79^ (2014)	United States	Supplemental Nutrition Assistance Program	ECEC	ECEC staff	Evaluation
Willis et al^80^ (2013)	United Kingdom	HENRY	ECEC	Health nurse	Evaluation
Willis et al^81^ (2016)	United Kingdom	HENRY	ECEC	Health nurse	Evaluation
Yin et al^82^ (2012)	United States	Miranos!	ECEC	ECEC staff	Design
Yin et al^83^ (2019)	United States and China	Miranos!	ECEC	ECEC staff	Evaluation
Study	Country	Intervention	Setting(s)	Professional(s)	Type of Study
Summary score (frequency)	United States: 30 United Kingdom: 8 Other: 5		Clinic: 9 Community: 7 ECEC: 16 Home: 13 Online: 1 Telephone: 3 Unknown: 2	Coach: 4 Dietician: 2 ECEC staff: 6 Health nurse: 10 Pediatrician: 2 Psychologist: 1 Unknown: 18	Design: 13 Evaluation: 28

^a^Ellipses indicate not reported, could not be coded; setting(s) and professional(s): ECEC, early childhood education and care; summary scores may not add up to total of 41 (100%) due to multiple options per study.

**TABLE 2.** Overview of Interventions: Cultural Adaptation and Food-Related Parenting Components

Intervention	Cultural Adaptation	Parenting Components
5-4-3-2-1-0 Program	Profile: 1: 30%, 2: 33%, 3: 83%, 4: 40%, 5%: 17%, 6: 0%	Self-efficacy, structure, enjoying healthy lifestyle
ANDALE Pittsburgh	Profile: 1: 50%, 2: 78%, 3: 0%, 4: 0%, 5: 14%, 6: 35%	Self-efficacy, being role model
Born in Brandfords Better start	Profile: 1: 0%, 2: 22%, 3: 0%, 4: 0%, 5: 0%, 6: 0%	...
CCC	Profile: 1: 0%, 2: 0%, 3: 0%, 4: 0%, 5: 0%, 6: 0%	...
CHL Program	Profile: 1: 50%, 2: 67%, 3: 0%, 4: 0%,5: 0%, 6: 0%	...
COACH	Profile: 1: 50%, 2: 22%, 3: 0%, 4: 0%, 5: 17%, 6: 75%	...
Communities for Healthy Living	Profile: 1: 50%, 2: 89%,3: 0%, 4: 20%, 5: 17%, 6: 0%	...
COOT Program	Profile: 1: 20%, 2: 0%, 3: 33%, 4: 0%, 5: 17%, 6: 0%	...
ELI Clinic	Profile: 1: 0%, 2: 11%, 3: 0%, 4: 0%, 5: 0%, 6: 0%	...
Family Check-Up	Profile: 1: 10%, 2: 0%, 3: 0%, 4: 0%, 5: 0%, 6: 0%	Structure, rewarding
FLAIR	Profile: 1: 10%, 2: 11%, 3: 50%, 4: 0%, 5: 17%, 6: 0%	Self-efficacy, structure
FSN	Profile: 1: 20%, 2: 56%, 3: 0%, 4: 20%, 5: 0%, 6: 0%	Self-efficacy, structure, being role model
GROW	Profile: 1: 0%, 2: 0%, 3: 0%, 4: 20%, 5: 17%, 6: 100%	Self-efficacy, structure, being role model, explaining to child, enjoying healthy lifestyle
HABITS	Profile: 1: 50%, 2: 11%, 3: 0%, 4: 60%, 5: 0%, 6: 25%	Self-efficacy, structure
HC2	Profile: 1: 90%, 2: 11%, 3: 0%, 4: 0%, 5: 50%, 6: 0%	Self-efficacy, being role model, enjoying healthy lifestyle
Healthy Habits, Happy Homes	Profile: 1: 0%, 2: 0%, 3: 0%, 4: 0%, 5: 0%, 6: 0%	Rewarding, being a role model
HHHF	Profile: 1: 10%, 2: 11%, 3: 0%, 4: 60%, 5: 100%, 6: 0%	Being role model
HENRY	Profile: 1: 10%, 2: 0%, 3: 0%, 4: 0%, 5: 0%, 6: 25%	Self-efficacy, structure, being role model
Hip-Hop to Health	Profile: 1: 0%, 2: 11%, 3: 0%, 4: 0%, 5: 0%, 6: 25%	Self-efficacy, enjoying healthy lifestyle
IDEFICS	Profile: 1: 0%, 2: 0%, 3: 0%, 4: 0%, 5: 0%, 6: 0%	...
Kids Go For Your Life	Profile: 1: 0%, 2: 0%, 3: 0%, 4: 0%, 5: 0%, 6: 0%	...
Miranos!	Profile: 1: 80%, 2: 89%, 3: 0%, 4: 20%, 5: 50%, 6: 25%	Self-efficacy, enjoying healthy lifestyle
More and Less ML	Profile: 1: 0%, 2: 0%, 3: 0%, 4: 0%, 5: 0%, 6: 0%	Self-efficacy, structure
NAP SACC intervention + Raising Healthy Kids	Profile: 1: 0%, 2: 0%, 3: 0%, 4: 0%, 5: 0%, 6: 0%	...
NET-works	Profile: 1: 0%, 2: 0%, 3: 0%, 4: 0%, 5: 0%, 6: 0%	Structure, rewarding, being role model
PaT(E)	Profile: 1: 0%, 2: 0%, 3: 0%, 4: 0%, 5: 0%, 6: 0%	...
Program study Hacioglu and Simsek^56^ (2019)	Profile: 1: 0%, 2: 0%, 3: 0%, 4: 0%, 5: 0%, 6: 0%	...
Program study McGarvey et al^63^ (2006)	Profile: 1: 10%, 2: 67%, 3: 33%, 4: 0%, 5: 0%, 6: 0%	Self-efficacy, structure, rewarding
Steps up to Growing Healthy	Profile: 1: 0%, 2: 0%, 3: 0%, 4: 0%, 5: 0%, 6: 0%	...
Supplemental Nutrition Assistance Program	Profile: 1: 20%, 2: 0%, 3: 0%, 4: 0%, 5: 0%, 6: 0%	...
Vida Saludable	Profile: 1: 25%, 2: 88%, 3: 0%, 4: 0%, 5: 0%, 6: 0%	Being role model
Summary scores	Profile: 1: 17.3% (SD = 25.5, range: 0-91), 2: 21.5% (30.8, 0-89), 3: 6.5% (18.6, 0-83), 4: 10.8% (21.3, 0-83), 5: 11.3% (22.5, 0-100), 6: 10.5% (23.1, 0-100) Overall (average profile: 1-6): 13.0% (SD = 13.7, range: 0-44)	

We analyzed the coded interventions with a categorical principal component analysis, which resulted in a solution with 6 internally consistent factors. Each factor reveals a different approach of cultural adaptation. The factors together “explained” 66% of the total variance (see also Supplemental Digital Content Appendix 3, available at: http://links.lww.com/FCH/A70).

Profile 1 is characterized by an integrated perspective on cultural diversity (Cronbach α = .88, eigenvalue λ = 6.03, “explained” variance: 14.0%). Considering the cultural background of the focus community plays a role in various phases of program development. The cultural adaptation often pertains to the surface structure in the development, implementation, and evaluation of interventions. Associated variables for this profile were translation of materials, adapting the language for the focus community during program development, taking into account parents' educational level, and racial and language matching in the implementation phase. Therefore, profile 1 mostly pertains to surface structure changes^18^ and cognitive-information processing.^20^

Profile 2 is characterized by an inclusive, collaborative model with a focus on deep structure (α = .88, λ = 5.99, variance: 13.9%). This profile describes interventions that focus on close cooperation with members of the focus community in the formulation of goals derived from the logic model during the design of the intervention, its production, and the evaluation of program effects. An associated variable for this profile was a selection of culturally appropriate program themes and components (ie, cultural values, traditions, customs), which is an example of cultural adaptation of the deep structure level of programs, related to the affective-motivational dimension.^20^

Profile 3 describes interventions in which attention is paid to cultural diversity during implementation and process evaluation (α = .82, λ = 4.84, variance: 11.2%). Associated variables for this profile were screening of staff related to inclusion competencies, complemented with a focus in the process evaluation on cultural subgroups and the selection of culturally appropriate measures. This profile is thus characterized by a focus on gathering experiences from the focus group during implementation and evaluation.

Profile 4 is characterized by a broad approach to cultural adaptation, based on a culture-specific logic model with a choice of culture-specific determinants in the logic model and the method of change (α = .84, λ = 4.52, variance: 10.5%). This culture-specific approach in the design is supplemented by collaboration with participants within the focus community during implementation and joint process evaluation.

Profile 5 characterizes studies with a focus on a logic model of change (α = .82, λ = 3.71, variance: 9.2%). For this profile, cultural adaptation involves multiple elements from a culturally appropriate logic model of change and involves culture-specific resources and a selection of culture-specific behavioral outcomes. These elements are complemented with the training of staff related to inclusion and a focus on acculturation in implementation.

Finally, the sixth profile describes interventions with a key focus on the use of networks in the local community as an implementation strategy (α = .73, λ = 3.27, variance: 7.6%). In this profile, a focus on geographical barriers (ie, distance to the family center, lack of transport) is complemented with close collaboration with the community, local centers or self-organizations (eg, church), and parent-to-parent recruitment. This focus aims to increase awareness in the community of the availability of the program and to stimulate families' entry in the program as first and vital steps of implementation in the community.^20^

We explored whether a different analysis with a smaller number of factors would result into different outcomes. Because our original analysis resulted in more homogeneous factors, we preferred this solution for analytical purposes.

### Intervention links with cultural diversity

Across the 6 profiles, the average CA score is 13% but with clear differences between the interventions (SD = 13.7, range = 0-44). Based on the factor structure identified, a total CA score has been determined on the basis of the different variables corresponding with each profile. These percentages show, at profile level, that the interventions vary greatly in the extent to which CA is explicitly addressed in the studies (see Table 2).

### Intervention links with parenting approach

The average PA score for the interventions is 14.5% (SD = 12.7, range = 0-39%); reports provided information related to the parenting approach for 21 interventions. These interventions link with the parenting context in the home situation by focusing on strengthening specific parenting skills. For example, some interventions aimed to increase parental self-efficacy in diet-related activities (40%) by having them “practice” certain skills as homework, or they may support parents in establishing rules and structures (33%, eg, eating at set times and/or at the table), or make them aware that they serve as role models for their child (30%). A family-centered approach was most common in our sample.

### Relationship between cultural adaptation and study characteristics

The CA and PA scores were positively correlated (*r*_s_ = 0.497, *P* = .004). As expected, we found higher scores for interventions with a family-centered perspective in which the parents' point of view plays a major role (*M* = 25.2, SD = 10.5, N = 8) than for programs without this focus (*M* = 8.7, SD = 12.2, N = 23), as the median test showed, χ^2^ = 11.5, *df* = 1, *P* = .003. CA scores were also higher for interventions in which empowerment explicitly plays a role than for other interventions (*M* = 24.0, SD = 13.7, *N* = 10 vs *M* = 7.7, SD = 10.4, N = 21, respectively), χ^2^ = 5.91, *df* = 1, *P* = .041.

## DISCUSSION

In our review study, we explored how studies describing different lifestyle interventions take into account the cultural diversity of families with young children. The included studies generally provided relatively little information related to the cultural adaptation or parenting style in the objectives, design, production, implementation, or evaluation of the interventions, acknowledging variation among studies. Our review suggests that cultural adaptation and parenting are not prominent themes in the literature,^8^,^9^ acknowledging that interventions do not need to include all elements that are distinguished in our conceptual framework.

Our analysis revealed 6 empirical profiles of cultural adaptation: an integrated perspective of cultural diversity with a focus on surface structure; an inclusive, collaborative model with a focus on deep structure characteristics; a focus on cultural diversity during implementation and process evaluation; cultural adaptation, based on a culture-specific logic model; logic model-driven implementation; and, finally, the use of networks in the local community for implementation. The identified empirical profiles were related to theoretical distinctions from the literature, including IM,^23^ family interventions,^40^ cultural adaptation, and prevention science.^10^,^17^

Our framework distinguishes various concrete actions to shape cultural adaptation across all phases of program development. Future program developers may use an integrated approach to the development of future programs (perhaps similar to the first profile), but it is also possible to complement a more restricted focus on cultural adaptation from a specific profile with other profiles to incorporate adaptation into the development of interventions in a structured fashion, because the identified profiles are independent and complementary.

Our analysis shows that the scope of cultural adaptation varies significantly among the included programs. This variation in scope explained much of the observed differences and suggests that cultural adaptation may be stimulated by integrating this in the full process of program development. Our empirical profiles suggest more specific strategies. Close collaboration with 1 or more cultural groups was related to changes at deep level, and it seems, therefore, valuable to include stakeholders in the design of the program to avoid a restricted focus on cultural adaptation with mostly changes at surface level.

Our findings showed that a focus on parenting styles and the empowerment of families in interventions are positively related to the cultural adaptation of interventions. Cultural adaptation was also positively associated with a family-centered approach, whereby the intervention is tailored to the needs of a family.^40^ These findings suggest that a culturally sensitive approach is also compatible with an empowering family approach.

### Limitations

Although cultural adaptation is an explicit theme in the studies we reviewed, it is possible that such adaptation of interventions occurs more frequently than is apparent from the scientific report.^9^ Not only may adaptations during the design phase not have been captured adequately in our study but also implementation practice could be more sensitive to the multicultural diversity and the parenting context of different families than is apparent from the scientific reports. Our results may therefore be conservative estimates. It should also be noted that the original studies were the input for our review and we coded explicit statements related to cultural adaptation from the authors in their reports.

The current review involves the first application of a new framework to analyze lifestyle interventions. Additional evaluation of intercoder agreement is needed for further validation.

## IMPLICATIONS FOR RESEARCH AND PRACTICE

The cultural adaptation and parenting approach may be strengthened in healthy lifestyle interventions to promote inclusive programs in practice. Our conceptual framework and the empirical profiles offer a tool for charting and strengthening the cultural adaptation and parenting approach of (future) interventions. Cultural fit may be improved by including individual elements for each step of program development without ticking all the boxes from our conceptual model. Combining the empirical profiles may also result into a hybrid, more comprehensive strategy to improve the cultural fit of lifestyle interventions for various families with children in their early years.

The first results of our psychometric analysis are promising, but further validation of our new measure is needed. In a next step of development and validation, it is important to investigate whether cultural adaptation and PA scores from our procedure are related to the successful implementation and positive effects of healthy lifestyle interventions from the literature. It is yet an open question whether the identified profiles may, for example, predict families' acceptance of the program, their adherence to the intervention, and/or a changes at child or parent level related to health knowledge, attitude, and behavior. In a next phase of development, we therefore aim to link the profiles of cultural adaptation with implementation and outcome measures from published studies, acknowledging the often modest level of cultural adaptation.^8^,^9^ This line of research may ultimately point out concrete, evidence-based strategies for successful cultural adaptation of healthy lifestyle programs and positive outcomes at child, parent, and family levels.

## Supplementary Material
