# Supplementary material for: Profiles of Cultural Adaptation and Parenting Approach for Childhood Obesity in Lifestyle Interventions for Families With Young Children: A Systematic Review
Source: Fam Community Health. 2024 Feb 19;47(2):95–107. doi: 10.1097/FCH.0000000000000397 (PMC10916755; doi:10.1097/FCH.0000000000000397)
Supplement: Supplementary file 1 [file fache-47-95-s001.docx]

Appendix 1

Search Profile for the Literature Review

| Domain | Key words |
| --- | --- |
| 1. Primary target group/setting | parent* or mother* or father* or "care giver*" or caregiver* or famil* or home* or "extended family" or communit* OR neighbourhood* |
| 1. Secondary target group | Toddler* or pre-school* or "pre-school child*" or "1-4 year*" or youngster* or child* |
| 1. Program | intervention* or prevention or program* or "intervention program" or "prevention program*" or "parental support" or treatment* or "famil* program" or "famil* intervention" or "communit* program" or "communit* intervention" or "neighborhood* program" or "neighborhood* intervention" or "program component*" or "program element*" or "intervention component*" or "intervention element*" |
| 1. Culture | "ecologic* valid*" or culture-sensitive or cultural-sensitive "cultur* sensitive" or "cultur* responsive" or "cultur* tailor*" or "social context*" or "cultur* care" or "family feedback" or family-cent* or strengths-based or assets-based or "funds of knowledge" or "cultur* valid" or co-construction or minorit* or ethnic* or ethno-cultur* or "cultur* belief*" or biocultural* or "cultur* adapt*" |
| 1. General parenting | "parenting style*" or parenting or "parent* behav*" or "parent* belief*" or "parent* practice*" or "child rearing" or "family culture" or "home culture" or "parent* socialization" or parent-child* or mother-child* or father-child* or "authoritarian parenting" or "authoritative parenting" or "democratic parenting" or "permissive parenting" or "neglectful parenting" or "parent* ethno-theor*" or "parent* treatment" or "parental conception*" or "parental misconception*" or "parent* philosoph*" or Baumrind or "Maccoby and Martin" or "cognitive belief*" |
| 1. General lifestyle | "healthy lifestyle*" or "unhealthy lifestyle*" or "healthy weight" or "unhealthy weight" or "weight gain" or "energy balance" or obes* or overweight or underweight or "BMI" or "body mass index" or "healthy behavio*" or "unhealthy behavio*" or "healthy lifestyle belief*" or "BMI z-score" or "BMI percentile" or "percent overweight" or adiposity |
| 1. Diet | "eating habit*" or diet* or nutrition or "child nutrition" or "healthy eating" or "healthy food*" or "unhealthy eating" or "unhealthy food*" or "energy intake" or "food culture" or calor* or cook* or meal* or culinar* or gastronom* or food* or "cultural variation" or feeding or "feeding pattern*" or "dietary behavio*" or "food practice*" or "feeding practice*" or "food custom*" or "dietary habit*" or "eating ritual*" or "life-span ritual*" or "folk health practice*" or "feeding interaction*" or "feeding pattern*" or "picky eating" or "taste development" or "dietary guidelines" or "food preparation strateg*" |
| Search profile | #1-2-3-4-5-6-7 |
