# Supplementary material for: Profiles of Cultural Adaptation and Parenting Approach for Childhood Obesity in Lifestyle Interventions for Families With Young Children: A Systematic Review
Source: Fam Community Health. 2024 Feb 19;47(2):95–107. doi: 10.1097/FCH.0000000000000397 (PMC10916755; doi:10.1097/FCH.0000000000000397)
Supplement: Supplementary file 2 [file fache-47-95-s002.docx]

Appendix 2:

Selection of Coding Scheme: Variables Related to Cultural Adaptation for each Intervention Mapping Step

| (1) **Logic model (design paper)** | | Page |
| --- | --- | --- |
| Special planning group with community informants / advisory board | ❑ yes |  |
| External a priori needs analysis for specific group (check goals) | ❑ yes |  |
| Description of intervention context: population / setting / community | ❑ yes |  |
| Program goals are tailored (‘framing’) to cultural group (Bernal..., 2006) | ❑ yes |  |
| Other | ❑ yes |  |
| Which: | | |

| (2) **Logic model of change (design paper) -** <https://ingbrief.wordpress.com/2018/06/18/program-logic-models-and-theory-of-change-kellogg-foundation-2004/> | | Page |
| --- | --- | --- |
| Outcomes for behaviour of cultural group/environment are specifie | ❑ yes |  |
| Selection of culturally appropriate determinants for lifestyle/food | ❑ yes |  |
| Matrix of change with culture-specific resources that promote or discourage healthy lifestyle   1. Any of the barriers distinguished by Barton (2003) – see below 2. Funds of knowledge, folk health beliefs | ❑ yes |  |
| Logic model of change is culturally appropriate (ecological validity) | ❑ yes |  |
| Other | ❑ yes |  |
| Which: | | |

| (3) **Program design (design paper)** | | Page |
| --- | --- | --- |
| Program themes, components, etc. are culturally appropriate (values, traditions, customs) | ❑ yes |  |
| Co-construction of intervention with stakeholders | ❑ yes |  |
| Choice of culturally appropriate & evidence-based change method  (see also Hennessy ea 2019) 🡪 may be related to logic model of change | ❑ yes |  |
| Selection/design of practical application to deliver program   1. Translation of materials in other (non-dominant) languages 2. Adapting language (not translation) 3. Formats: pictures, games, etc. | ❑ yes  ❑ yes  ❑ yes  ❑ yes |  |
| Other | ❑ yes |  |
| Which: | | |

| (4) **Program production (pilot paper)** | | Page |
| --- | --- | --- |
| Production, pilot, formative evaluation, refining materials in culturally responsive way | ❑ yes |  |
| Other | ❑ yes |  |
| Which: | | |

| (5) **Program implementation plan (pilot and evaluation papers)** | | Page |
| --- | --- | --- |
| Identification of program users (clients) in cultural group   1. Identification of potential program user (implementer, adopter, maintainer) in cultural group 2. Extended family as point-of-entry 3. Guidance: collaboration with community, local center, self-organisation (church, other) 4. Guidance: peer-to-peer (parent-to-parent, ‘snowballing’) | ❑ yes  ❑ yes  ❑ yes  ❑ yes  ❑ yes |  |
| Design of culturally sensitive implementation:   1. Screening of staff related to inclusion competencies 2. Training (pre or in-service) staff related to inclusion 3. Matching practitioner-family: language matching (Bernal & Siaz-Santiago, 2006) 4. Matching practitioner-family: racial matching (Bernal & Siaz- Santiago, 2006) | ❑ yes  ❑ yes  ❑ yes  ❑ yes  ❑ yes |  |
| Outcomes for program use are stated with reference to cultural group | ❑ yes |  |
| Barton’s (2003) barriers (b) to access intervention taken into account for cultural group (and based on framework Bernal & Siaz-Santiago, 2006):   1. Geographical: distance to center, transport 2. Physical: handicap 3. Temporal: time 4. Financial: poverty/enough resources 5. Socio-cultural: education level 6. Socio-cultural: language / literacy 7. Socio-cultural: acculturation 8. Socio-cultural: inclusion/exclusion, network, support system 9. Socio-cultural: stigma/positive discrimination? 10. Socio-cultural: relationship with country/culture of origin | ❑ yes  ❑ yes  ❑ yes  ❑ yes  ❑ yes  ❑ yes  ❑ yes  ❑ yes  ❑ yes  ❑ yes |  |
| Other | ❑ yes |  |
| Which: | | |

| (6) **Evaluation plan (pilot and evaluation papers)** | | Page |
| --- | --- | --- |
| Process evaluation is tailored to cultural group | ❑ yes |  |
| Selection of culturally appropriate measures for process evaluation | ❑ yes |  |
| Co-evaluation of process with stakeholders (e.g., diversity audit) | ❑ yes |  |
| Process evaluation with focus on (one or more) cultural subgroups | ❑ yes |  |
| Effect evaluation is tailored to cultural group | ❑ yes |  |
| Selection of culturally appropriate measures for effect evaluation | ❑ yes |  |
| Co-evaluation of process with stakeholders (e.g., diversity audit) | ❑ yes |  |
| Effect evaluation with focus on (one or more) cultural subgroups | ❑ yes |  |
| Effect evaluation includes cultural moderator | ❑ yes |  |
| Effect evaluation includes cultural mediator | ❑ yes |  |
| Other | ❑ yes |  |
| Which: | | |
