# Supplementary material for: Profiles of Cultural Adaptation and Parenting Approach for Childhood Obesity in Lifestyle Interventions for Families With Young Children: A Systematic Review
Source: Fam Community Health. 2024 Feb 19;47(2):95–107. doi: 10.1097/FCH.0000000000000397 (PMC10916755; doi:10.1097/FCH.0000000000000397)
Supplement: Supplementary file 3 [file fache-47-95-s003.docx]

Appendix 3:

Factor Loadings for the Six Dimensions of Cultural Adaptation

| Variable | Dimension | | | | | |
| --- | --- | --- | --- | --- | --- | --- |
|  | 1 | 2 | 3 | 4 | 5 | 6 |
| IM5.4.matching_racial | .906 | .019 | -.119 | .089 | .022 | -.178 |
| IM5.5.education | .822 | .102 | -.111 | .462 | -.060 | -.073 |
| IM5.6.language | .731 | .204 | .310 | .288 | .009 | .314 |
| IM6moderator | .713 | .069 | -.084 | -.273 | .059 | .158 |
| IM6mediator | .701 | -.196 | -.017 | -.176 | .191 | -.204 |
| IM5implementation | .641 | .336 | -.059 | .354 | .064 | .040 |
| IM3translation | .525 | .453 | .352 | -.021 | .078 | .258 |
| IM3materials | .485 | .401 | .294 | -.175 | .230 | -.015 |
| IM3adapting | .480 | .232 | -.074 | .153 | .132 | .439 |
| IM1program_goals | .479 | .225 | -.146 | .169 | -.131 | .200 |
| IM5.4.financial | .467 | .078 | .035 | .428 | .403 | .020 |
| IM1check_goals | .082 | .907 | -.046 | .193 | .058 | -.044 |
| IM1advisory_board | .075 | .886 | -.118 | .124 | -.007 | .126 |
| IM3components | .110 | .787 | .263 | .141 | -.052 | .210 |
| IM1description_population | .076 | .757 | -.053 | -.232 | .127 | -.093 |
| IM4production | -.045 | .719 | .367 | .328 | .382 | -.112 |
| IM6co_evaluation_effect | .150 | .660 | -.115 | .402 | -.068 | -.139 |
| IM6effect | .104 | .596 | -.150 | -.070 | .197 | .032 |
| IM3stakeholders | .027 | .580 | -.206 | .576 | -.053 | -.042 |
| IM3formats | .396 | .470 | .384 | -.099 | .074 | -.328 |
| IM5.1.screening | .020 | -.001 | .865 | .074 | -.040 | .013 |
| IM6process | -.051 | -.055 | .833 | .020 | .006 | -.033 |
| IM6subgroups_process | -.005 | -.143 | .827 | .031 | -.015 | -.012 |
| IM6measures_process | .059 | -.117 | .784 | .070 | -.053 | .028 |
| IM5.10.origin | -.057 | .253 | .754 | -.083 | -.087 | -.105 |
| IM5.9.stigma | -.137 | .199 | .344 | -.168 | -.023 | -.156 |
| IM6measures_effect | .097 | .125 | -.215 | .072 | -.015 | .074 |
| IM5.8.inclusion | .137 | -.108 | -.117 | .873 | .258 | .000 |
| IM2determinants | .131 | -.095 | .353 | .809 | .207 | .007 |
| IM6co_evaluation_process | .043 | .161 | -.012 | .729 | -.150 | .161 |
| IM5program_users | -.151 | .239 | -.096 | .641 | .361 | -.058 |
| IM5.1.identification | .099 | .155 | .316 | .472 | .365 | .275 |
| IM3change_method | .144 | .163 | -.174 | .364 | -.217 | -.276 |
| IM2ecological_validity | -.111 | .165 | -.073 | .109 | .847 | .180 |
| IM5.7.acculturation | .194 | .362 | -.078 | .022 | .766 | .144 |
| IM2outcomes | .395 | -.280 | -.035 | .085 | .741 | -.200 |
| IM5.2.training | .615 | -.008 | -.047 | .002 | .678 | -.171 |
| IM2matrix | -.206 | .130 | -.088 | .260 | .573 | -.267 |
| IM5.3.matching_lang | .426 | .032 | .451 | -.097 | .524 | .456 |
| IM5.4.guidance_peer | .045 | .200 | -.087 | -.182 | .142 | .882 |
| IM5.3.guidance_comm | -.015 | -.052 | -.094 | .266 | -.189 | .690 |
| IM6subgroups_effect | -.092 | -.141 | -.034 | -.042 | .028 | .601 |
| IM5.1.geographical | .472 | .074 | -.136 | .086 | -.134 | .557 |

*Note*: The names of the variable indicate the IM step and a label for each variable from the coding scheme.
